# Interventions to reduce falls in hospitals: a systematic review and meta-analysis

**DOI:** 10.1093/ageing/afac077

**Published:** 2022-05-06

**Authors:** Meg E Morris, Kate Webster, Cathy Jones, Anne-Marie Hill, Terry Haines, Steven McPhail, Debra Kiegaldie, Susan Slade, Dana Jazayeri, Hazel Heng, Ronald Shorr, Leeanne Carey, Anna Barker, Ian Cameron

**Affiliations:** 1 La Trobe University Academic and Research Collaborative in Health, Melbourne, Victoria, Australia; 2 The Victorian Rehabilitation Centre, Healthscope, Glen Waverley, Victoria, Australia; 3 School of Allied Health, Human Services and Sport, La Trobe University, Melbourne, Australia; 4 Western Australian Centre for Health & Ageing, School of Allied Health, The University of Western Australia, Perth, Western Australia, Australia; 5 School of Primary and Allied Health Care, Monash University, Melbourne, Victoria, Australia; 6 Australian Centre for Health Services Innovation and Centre for Healthcare Transformation, School of Public Health & Social Work, Queensland University of Technology, Brisbane, Queensland, Australia Australia; 7 Digital Health and Informatics Directorate, Metro South Health, Brisbane, Queensland, Australia; 8 Holmesglen Institute and Monash University, Melbourne, Victoria, Australia; 9 Geriatric Research Education and Clinical Center, Malcom Randall VAMC, Department of Epidemiology, University of Florida, Gainesville, FL, USA; 10 Department of Epidemiology, University of Florida, Gainesville, FL, USA; 11 Florey Institute of Neuroscience and Mental Health, Melbourne, Victoria, Australia; 12 Silver Chain, Melbourne, Victoria, Australia; 13 John Walsh Centre for Rehabilitation Research, Northern Sydney Local Health District and The University of Sydney, Sydney, NSW, Australia

**Keywords:** falls, hospital, physiotherapy, prevention, education, exercise, older people, systematic review

## Abstract

**Background:**

Falls remain a common and debilitating problem in hospitals worldwide. The aim of this study was to investigate the effects of falls prevention interventions on falls rates and the risk of falling in hospital.

**Design:**

Systematic review and meta-analysis.

**Participants:**

Hospitalised adults.

**Intervention:**

Prevention methods included staff and patient education, environmental modifications, assistive devices, policies and systems, rehabilitation, medication management and management of cognitive impairment. We evaluated single and multi-factorial approaches.

**Outcome measures:**

Falls rate ratios (rate ratio: RaR) and falls risk, as defined by the odds of being a faller in the intervention compared to control group (odds ratio: OR).

**Results:**

There were 43 studies that satisfied the systematic review criteria and 23 were included in meta-analyses. There was marked heterogeneity in intervention methods and study designs. The only intervention that yielded a significant result in the meta-analysis was education, with a reduction in falls rates (RaR = 0.70 [0.51–0.96], *P* = 0.03) and the odds of falling (OR = 0.62 [0.47–0.83], *P* = 0.001). The patient and staff education studies in the meta-analysis were of high quality on the GRADE tool. Individual trials in the systematic review showed evidence for clinician education, some multi-factorial interventions, select rehabilitation therapies, and systems, with low to moderate risk of bias.

**Conclusion:**

Patient and staff education can reduce hospital falls. Multi-factorial interventions had a tendency towards producing a positive impact. Chair alarms, bed alarms, wearable sensors and use of scored risk assessment tools were not associated with significant fall reductions.

## Key Points

- Falls in hospitals can be prevented with evidence-based patient education.
- Health professional education about falls prevention can also reduce the rate of falls and fall-related injuries.
- Significant falls reduction in hospitals did not occur with bed or chair alarms, or use of sensors.

## Introduction

Hospital falls remain a problem worldwide, despite sustained falls prevention efforts in public and private healthcare settings [1, 2]. Falls rates, which are usually expressed per 1,000 bed days, typically range from 2 to 8 in acute hospitals, geriatric wards and emergency [3–5]. In rehabilitation hospitals where patients are encouraged to mobilise, falls rates typically range from 3 to 16 per 1,000 bed days [6, 7]. Injuries occur in around 30% of hospital falls [8, 9]. There can be minor injuries such as lacerations, contusions, sprains and strains as well as more serious injuries such as head injuries, fractures and death [8–10]. Anxiety and fear of falling can also arise [11].

Hospital falls prevention strategies include patient education, clinician education, environmental adaptations, the use of assistive devices, therapeutic exercises, medication reviews, optimal nutrition, management of cognitive impairment and falls mitigation policies, systems and leadership [1, 12, 13]. Some examples of systems include post-fall team ‘huddles’ [14], falls reports at nursing handover [15], auditing [16] and reporting monthly falls [12]. Some of these interventions have been investigated in isolation eg. [14, 15]. Others have been evaluated as part of a multi-factorial approach to mitigating hospital falls eg. [1, 17].

Patients do not always realise their risk of falling whilst in hospital [12, 18, 19] even though people over the age of 65 years and those 50 years or older with two or more co-morbidities are at high risk [20]. A Cochrane review [1] published in 2018 found little robust evidence in support of hospital falls mitigation interventions and concluded that ‘multifactorial interventions may reduce the rate of falls, although this is more likely in a rehabilitation or geriatric ward setting (low quality evidence)’. As well as noting few clinical trials of high quality, the Cochrane review excluded patients with stroke and included care homes. Many new trials have been published in the last 4 years hence we conducted a new systematic review and meta-analysis of the hospital falls literature without mixing the results with aged care home settings. Uniquely, we also included an analysis of the effects of scored falls risk screening tools as these are often used as a fall mitigation strategy [5, 20].

The primary aim was to evaluate the effects of single- and multi-factorial interventions on falls rates and risk in hospitals, and to grade the strength of evidence and quality of the studies. The research question was: ‘What are the effects of falls prevention interventions on falls outcomes for adults in hospital settings [10]?’

## Methods

The design and conduct of this review and analysis was informed by principles in the Cochrane guidelines [21] and the Preferred Reporting Items for Systematic Reviews and Meta-Analyses (PRISMA) guidelines were followed. The review was prospectively registered on PROSPERO (2017: CRD 42017058887).

### Search strategy

The Medline, CINAHL, PsycInfo, Embase, AMED, PEDro and Cochrane electronic databases were searched from all records until 31 May 2021. Search terms were entered with three concepts: (i) falls (ii) hospital setting (iii) study design. Terms within each concept were combined with the OR Boolean operator, and the three concepts were combined with the AND Boolean operator. Each of the terms were then aligned to MeSH subject headings and then searched via keywords. Examples of terms used for concept 1 included ‘falls’, ‘fall prevention’ and ‘accidental fall’. Examples of terms used in concept 2 were ‘acute care’, ‘rehabilitation’, ‘hospital’, ‘hospitalisation’, ‘ward’ and ‘clinic’. Examples of terms used in concept 3 were ‘randomised controlled trial’ (RCT), ‘trial’, ‘controlled clinical trial’ and ‘study’. Journal contents pages and articles in press from medical and ageing journals were also hand searched, and reference lists were screened. Publication details were sent to bibliographic software and any duplicates were removed.

### Eligibility criteria

Studies were included in the systematic review if they: (i) were published in English; (ii) included a falls intervention in a hospital setting; (iii) investigated falls as a primary or secondary outcome and falls data were reported and (iv) included a contemporary comparison group (either as a RCT, quasi-randomised, cluster RCT, comparative study or quasi-experimental study). In the meta-analysis we only included studies for which the falls rate ratios and odds ratios (ORs) were provided by authors or could be calculated from the data reported. Conference proceedings, case studies, clinical commentaries, website reports and reviews were excluded. Studies were excluded if they only had paediatric samples or an historical comparison group or if they only reported falls in a list of adverse events.

The titles of manuscripts were first screened for eligibility by four researchers. Full abstracts were then independently screened by three pairs of reviewers. Any abstracts that failed to align with the inclusion criteria or had exclusions were removed. Complete text versions of the selected manuscripts were retrieved, and three pairs of reviewers independently analysed them. Discrepancies were discussed by our review team until consensus was reached.

### Outcomes and data extraction

The primary falls outcomes were (i) reduction in the rate of falls (falls per unit of person time, such as bed days (typically falls per 1,000 bed days)), designated as the rate ratio (RaR); (ii) reduction in falls risk, defined by the odds of being a faller in the intervention compared to control group (OR). For each article, we extracted the setting, inclusion and exclusion criteria, random allocation procedure, participant demographics and diagnosis, falls interventions and how falls were measured and recorded.

### Intervention category

Study interventions were grouped according to the following taxonomy: (i) a single intervention which was one of the following: (a) direct education of patients or clinicians; (b) environment modifications (e.g. flooring, lighting, ramps, signs); (c) assistive devices (e.g. call bell, alert bracelet, bed alarm, traction socks, walking frame, stick, chair assist, lowered bed, technologies); (d) systems, service models, social context, leadership, policies or procedures to prevent falls; (e) rehabilitation, physiotherapy, physical activities, or other therapeutic exercises delivered in hospital; (f) medication management; (g) dietary modification, including vitamins, or (ii) multi-factorial interventions which combined two or more approaches. Each individual study was grouped according to the most predominant intervention type, unless multiple intervention arms were specified. The taxonomy was a simplified version of that used in the prior Cochrane review [1]; we used the phrase ‘education’ rather than ‘knowledge interventions’ and environment/assistance was separated into environment modifications and assistive devices.

### Data synthesis

For the rate of falls, the RaR and 95% confidence intervals (CI) that were reported in each paper were used. If both adjusted and unadjusted rate ratios were reported, we preferentially used estimates that had been adjusted for clustering (if applicable) and if multiple estimates were available due to adjustment for co-variates, we included the one which the authors either specified a priori in the trial registration or highlighted in their study summary (abstract or conclusion). When not reported, we emailed authors to obtain the data.

To synthesise the risk of falling, we used ORs and reported 95% CI if available. If both adjusted and unadjusted estimates were reported we used the unadjusted estimate, unless the adjustment was for clustering (e.g. clustering of patients within sites). If an OR and 95% CI were not reported and appropriate data were available, we calculated an OR and 95% CI in Review Manager [22]. For these calculations, we used the number of participants randomised to each group. For cluster correcting, the Intra-cluster Correlation Coefficient (ICC) from Hill *et al.* [7] was used. The cluster correction was also applied for quasi-experimental designs. Thus, for Barker (2016) we had to calculate an OR for the fallers versus non-fallers analysis based on raw data, then apply a variance inflation factor. The Dykes (2010) study did not present an adjusted OR for the fallers versus non-fallers outcome (just an adjusted *P*-value) and we calculated this based on the raw data, adjusting the ICC by applying a variance inflation factor.

Within each intervention category the generic inverse variance method in Review Manager was used to run the meta-analysis and we created forest plots using R. This required entering the natural logarithm of the RaR or OR and standard error (SE) for each trial, which were calculated in Microsoft Excel [23].

### Risk of bias assessment

The included studies in the systematic review were assessed for risk of bias. The Cochrane Risk of Bias 2 Instrument was used to evaluate risk of bias for randomised or quasi-RCT [21]. The Cochrane Cluster Randomized Parallel Group Trials 2 Instrument was used for cluster randomised controlled trials [21].

Studies were assessed independently for risk of bias by three reviewers from our review team and consultation with a fourth reviewer. Risk of bias figures were prepared for the RCTs and a summary table was produced for the RCTs. We selected a valid instrument for other study designs, such as quasi-experimental designs, from the Joanna Briggs Institute (JBI) Critical Appraisal Tools database [24]. The reviewers assigned risk of bias as low, medium or high.

### GRADE strength of evidence

For each meta-analysis, the Grading of Recommendations, Assessment, Development and Evaluations (GRADE) instrument was used to appraise the strength of evidence for the falls rates and risk [25, 26]. Randomised trials were appraised separately from non-RCTs. Components assessed were study design, risk of bias, indirectness, imprecision and inconsistency.

### Consensus on exercise reporting template

Two reviewers independently extracted all exercise intervention component data from the included studies by using the Consensus on Exercise Reporting (CERT) checklist. The CERT has 16 categories and 19 separate items considered essential in the reporting of reproducible exercise interventions [27]. Each item was scored 1 (clearly reported) or 0 (not reported or not clearly described), and a score out of a total of 19 was calculated.

## Results

### Systematic review results

As shown in the PRISMA diagram (Supplementary File Figure 1), the electronic database search identified 11,186 studies; 26 were identified from the manual search of reference lists and relevant journals. After 3,006 duplicates were removed 8,206 articles remained as the total yield. From reviewing titles and abstracts 7,976 studies were excluded, and the full text of the remaining 230 studies were downloaded for detailed assessment. Of these 187 were excluded and 43 studies were included in the systematic review. Two additional studies assessing the efficacy of Falls Risk Assessment Tools (FRATs) as a fall mitigation strategy were also included [28, 29]. Supplementary File Table 1 summarises the characteristics of the included studies. Supplementary File Figures 2–3 and Supplementary File Tables 2–4 summarise the evaluations associated with the systematic review and meta-analysis, such as risk of bias, method quality, components of multi-factorial interventions, GRADE results and CERT results.

For the systematic review, multi-factorial interventions were evaluated in 16 studies [5, 30–44] (Supplementary File Table 1 and Table 4). Seven papers investigated the effect of providing assistive devices such as low beds, sensors and bed and chair alarms [45–51]. Five evaluated exercise or rehabilitation therapies [52–56]. Two evaluated the effects of patient education [7, 57] and one was on clinician education [7]. Ten investigations evaluated hospital systems, service models, policies and procedures to reduce falls [58–67]. Three investigated environmental modifications [52, 68, 69] and one analysed the effects of medication (Vitamin D prescription) [70].

### Meta-analysis results: single interventions

#### Education

**Figure 1:**
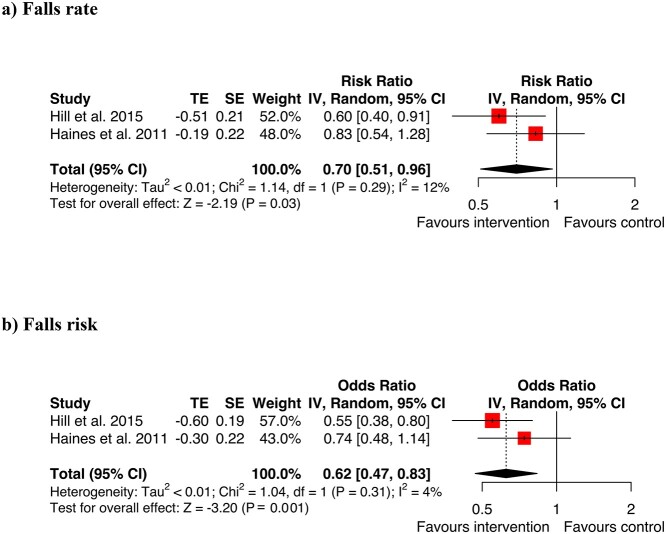
Meta-analysis education for (a) falls rate (b) falls risk.

Two trials assessed three different patient educational packages [7, 57] (Figure 1, Supplementary File Table 1). Haines et al. [57] analysed a comprehensive educational program and a version with only educational materials. As the comprehensive program was most like Hill [7], this package was used for the meta-analysis which showed positive results for both the odds of falling and rate of falls. The overall summary RaR was 0.70 [0.51–0.96], *Z* = −2.19, *P* = 0.03 (Figure 1). The overall summary OR was 0.62 [0.47–0.83], *Z* = −3.20, *P* = 0.001. The patient education studies in the meta-analysis were rated by GRADE as having a high strength of evidence (Supplementary File Table 5). The trial by Hill *et al.* [7] also incorporated staff education, showing beneficial effects on falls.

#### Assistive devices

**Figure 2:**
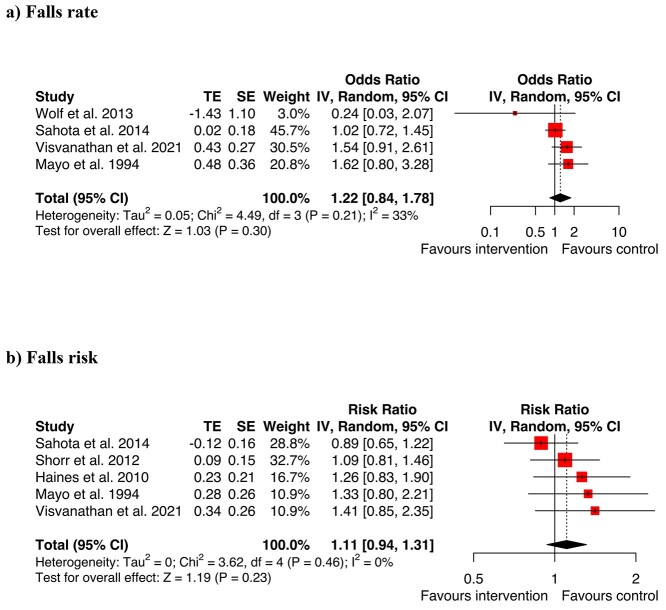
Meta-analysis assistive devices for (a) falls rate (b) falls risk.

Four studies included in the meta-analysis assessed the rate of falling associated with assistive devices and five assessed falls risk associated with devices such as bed alarms, wearable sensors and alert bracelets (Figure 2). The overall results for assistive devices showed no significant effects on rate of falls or odds of falling (RaR = 1.22, CI 0.84–1.78, *Z* = 1.03, *P* = 0.30; OR = 1.1, CI 0.94–1.31, *Z* = 1.19, *P* = 0.23). The study by Healey *et al.* [44] could not be included in the meta-analysis because effect sizes and CI for rate or odds of falls in hospitals alone were not reported. The study by Tideiksaar [49] *et al.* on bed alarms did not have effects sizes and CI reported in a way enabling inclusion in the meta-analysis. It had non-significant results and a high risk of bias when evaluated with the Cochrane risk of bias tool.

#### Rehabilitation and exercise therapies

**Figure 3:**
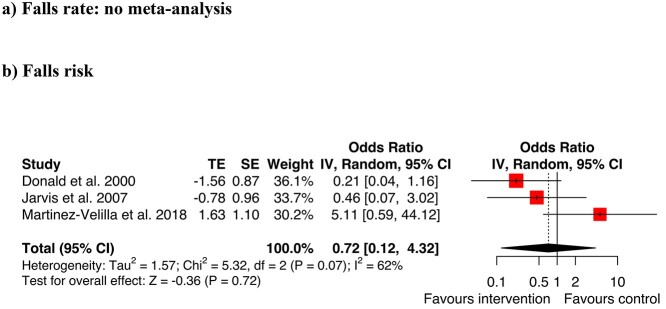
Meta-analysis additional rehabilitation for (a) falls rate (b) falls risk.

ORs for three studies were included in a meta-analysis [52, 53, 56]. The analysis did not show an overall reduction in falls risk with rehabilitation and exercise therapies (OR = 0.72, CI 0.12–4.32; *Z* = −0.36, *P* = 0.72, Figure 3). Only the rehabilitation study by Treacy [55] reported the RaR, with no significant change in the rate of falls (IRR:1.13, 95% CI 0.65–1.96, *P* = 0.662). The trial by Padula *et al.* [54] could not be included in this meta-analysis because effect sizes and CI for the rate of falls were not reported. It was of moderate quality, scoring 6/9 using the JBI quasi-experimental appraisal tool and reported similar falls rates between intervention and control groups.

#### Systems

The system-based falls mitigation interventions that were implemented varied in content and approach. The data from these nine studies were not pooled in a meta-analysis as effect sizes were not reported. The use of hourly rounding, bedside handover, electronic surveillance or a patient safety officer did not significantly reduce the rate of hospital falls in five studies [58, 60–62, 65]. In a trial involving medical review of patient falls, the falls rate per 1,000 patient days was 10.6 in the control group compared to 1.5 in the experimental group following medical review and accompanying actions. This difference was statistically significant (*P* < 0.004) [59].

Hardin *et al.* [63] reported a significant reduction in falls per 1,000 admissions between intervention (web cameras in patient rooms) and control groups. Despite favourable results, this paper was not able to be included in the meta-analysis because the effect sizes and CI for the rate of falls or odd of falls were not reported or available. It was rated as having some concerns for risk of bias. Bott *et al.* [64] reported a reduction in falls with the use of an embodied conversational agent. There was no mention of whether this was statistically significant, and the study had a medium risk of bias. Sheppard *et al.* [66] conducted a time series analysis on the effects of implementing the NICE falls prevention clinical guidelines. They found fewer falls per 1,000 occupied bed days in the intervention period (5.89) compared to the control period (6.62). This study had a high risk of bias (Supplementary File Table 2). Montejano-Lozoya *et al.* [67] assessed the effect of an educational intervention for hospital nurses. There was a higher incidence of falls in the control group compared with the intervention group although the sample size was comparatively low. The risk of bias for this study was high (Supplementary File Table 3).

#### Environmental modifications

**Figure 4:**
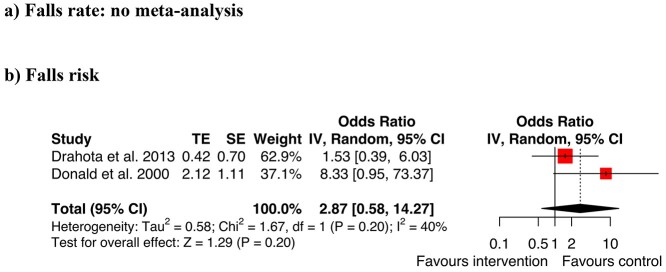
Meta-analysis environmental modifications for (a) falls rate (b) falls risk.

Two studies evaluated the outcomes of different types of flooring (Figure 4). Donald [52] investigated if carpet (intervention arm) was safer than vinyl (control arm). Drahota *et al.* [68] compared a thicker floor (intervention arm; 8.3 mm vinyl over a fibreglass mat) with standard flooring (control arm; 2 mm vinyl or thermoplastic tiles). Both reported results trending towards higher falls rates and risks for the intervention conditions. When combined in a summary meta-analysis, the overall difference between conditions was not statistically significant and the CIs were large (OR = 2.87, CI 0.58–14.27, *Z* = 1.29, *P* = 0.20). An investigation by Hanger [69] analysed the effects of low impact flooring and reported median falls rates. Because medians were used, the data could not be pooled with the other studies in the meta-analysis. Hanger [69] found a small and non-significant reduction in falls rates with low impact flooring.

#### Medications

One study, by Burleigh *et al.* [70] investigated the effects of delivering vitamin D plus calcium to hospitalised patients, compared with calcium alone. Although the number of fallers was less in the intervention group (*n* = 36) compared to the control group (*n* = 45) this reduction was not statistically significant (RR 0.82 CI 0.59–1.16). This article was rated as having a low risk of bias using the Cochrane risk of bias 2 tool (Supplementary File Figure 2).

### Meta-analysis results for multi-factorial interventions

**Figure 5:**
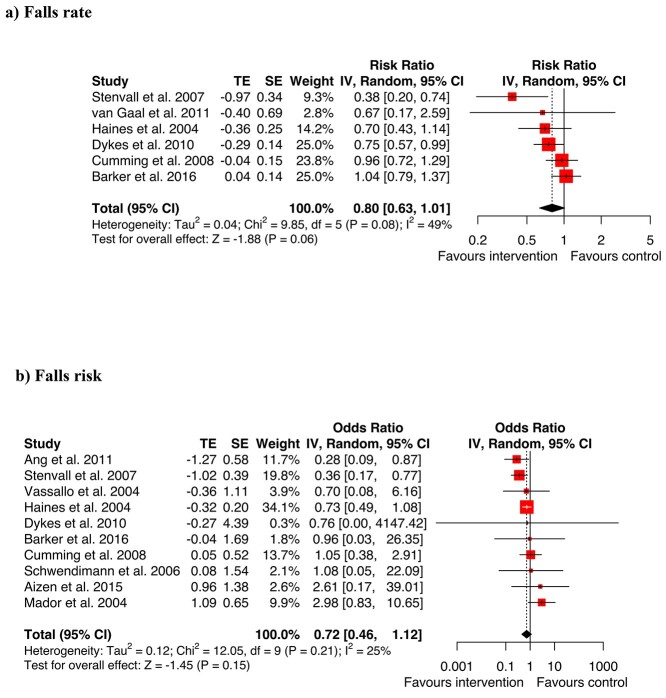
Meta-analysis multifactorial interventions for (a) falls rate (b) falls risk.

The meta-analysis of multi-factorial studies (Figure 5) showed a trend towards an overall reduction in the rate of hospital falls, although this did not quite reach statistical significance (RaR = 0.8, 95% CI 0.63–1.01, *Z* = −1.88, *P* = 0.06). The OR could be calculated for 10 of the multi-factorial studies [5, 30–34, 38–41]. Overall, the meta-analysis did not identify a statistically significant reduction in falls risk (OR = 0.72, CI 0.46–1.12, *Z* = −1.45, *P* = 0.15).

There were positive results for individual multi-factorial trials by Ang [31], Dykes [33], Haines [34], Stenvall [40] and Vassallo [41]. The multi-factorial falls prevention studies by Healey, Monro *et al.* [35] and Healey, Low *et al.* [44] also reported significant reductions in the risk of falls (Healey *et al.* 2004., *P* = 0.006); and in the rate of falls (Healey 2014: *P* = 0.01). The two Healey studies were excluded from the meta-analysis and forest plots because effect sizes and CIs for rate of falls or odds of falls in hospitals alone were not reported or available. Likewise, the multi-factorial studies by Koh [36] and Krauss [37] had non-significant results and were not included in the meta-analysis because OR or RaR could not be calculated. A multi-factorial falls prevention study by Wald [42] found no significant difference between intervention and control groups (4.8 versus 6.7 falls per 1,000 patient days, 95% CI −9.63 to 13.3). It was not able to be included in the meta-analysis because an estimate of the rate ratio effect was not reported or able to be calculated as the distributions were not available.

### Scored falls risk screening tools

Scored FRATs have been historically used in hospitals as another fall mitigation strategy. Clinicians typically assign FRAT scores out of 10 for each patient to guide the assignment of interventions based on the score. Despite their widespread use in the past, there is limited evidence of their efficacy. Two recent large RCTs assessed the effectiveness of these tools and found that removal of the risk scoring component of the FRAT did not result in inferior fall outcome [28, 29] (Supplementary File Table 6). Jellett *et al.* (2020) [28] conducted their stepped-wedge trial across nine hospital units across 10 months and found that the rate of falls was not impacted by divesting from the use of a scored FRAT and time was saved. Morris *et al.* (2021) [29] conducted a parallel cluster RCT across 10 hospitals over 3 months and found that removing the requirement to perform scored risk screening did not negatively impact fall rates.

### Risk of bias

Fifteen of the 43 included studies in the systematic review were RCTs. Risk of bias was assessed with the Cochrane Risk of Bias 2 Instrument [21] (Supplementary File Figure 2). For two of the studies, there were two trial arms and each arm was appraised separately [52, 57]. Many of the RCTs had a low risk of bias [31, 34, 40, 52, 55–57, 70]. Four were at medium risk of bias [38, 46, 47, 52]. There were concerns about the randomisation methods, deviations from the planned intervention in some studies, or issues with measurement of the outcomes. Some trials were assessed to have a high risk of bias, with concerns about randomisation, or protocol deviations [49, 50, 53, 59], or missing outcome data [49, 53]. Risk of bias for RCTs as a percentage is shown in Supplementary File Figure 3.

Thirteen of the included studies were cluster RCTs (cRCTs). For these, the risk of bias was assessed with the Cochrane Cluster Randomised Parallel Group Trials 2 Instrument [21] (Supplementary File Table 2). Six of the cRCTs had a low risk of bias [5, 7, 33, 43, 45, 48]. Six were rated as having ‘some concerns’ due to issues with randomisation [30, 44, 68], imbalance in participant characteristics [68], deviations from the intended interventions [30, 44, 68], missing outcome data [35], or problems with measurement of the outcome [32, 63]. One had a high risk of bias due to problems with randomisation, identification and recruitment of participants, deviations from the planned intervention, measurement issues and selection of reported results [66].

Fifteen of the included studies in the systematic review had a non-randomised design [36, 37, 39, 41, 42, 51, 54, 58, 60–62, 64, 65, 67, 69]. Supplementary Table 3 shows that many of these scored well on method quality [36, 37, 39, 41, 42, 51, 58, 60–62, 65, 69]. Three had a medium risk of bias; one associated with the statistical analysis, differences in participants in control and experimental groups and a lack of multiple measurements [54] another due to differences in participants, lack of follow-up and statistical analysis [64] and one had differences in participants in the control and experimental groups, uncertainty regarding multiple measurements, and unclear reporting of follow up measures [67]. The studies on divesting from scored FRAT screening both had a low risk of bias (Supplementary Table 6).

For the odds of falling, the GRADE showed strong evidence for education [7, 57] and low for assistive devices [46, 47] (Supplementary Table 5). The strength of evidence as rated using GRADE was very low for additional rehabilitation therapies [52, 53, 56], environmental modifications [52, 68], RCT multi-factorial interventions [5, 30–34, 38, 40] and non-RCT multi-factorial interventions [39, 41]. For the rate of falls, the strength of evidence was rated high by GRADE for education [7, 57] and moderate for assistive devices. It was very low for multi-factorial studies (Supplementary Table 5).

### CERT results

There were nine studies that included an exercise component to prevent hospital falls [32, 34, 39, 40, 52–56]. Supplementary Table 7 shows the CERT evaluation, with low comprehensiveness of exercise reporting overall. The mean was 8.6 (SD 4.3) out of a possible score of 19. Three studies had a CERT score greater than 10 [34, 55, 56]. Some required reference to previous studies, website links, or published protocols. One weblink to an exercise manual was inactive and the author provided the details [34]. Five well described CERT items were instructor qualifications (9/9 included studies), reporting of adverse events (9/9), the setting (9/9), supervision (8/9) and whether the exercise intervention was generic or tailored to individual needs (7/9). The poorly described elements were exercise equipment (3/9), individual or group delivery (4/9), exercise adherence (2/9), exercise motivation strategies (0/9), exercise progression (2/9), exercise details (4/9) and whether the exercise intervention was delivered as planned (3/9).

## Discussion

Falls in hospital are the most common safety incident affecting older people and are a frequent cause of concern for staff, complaints by families and sometimes coroner inquests or civil claims. Beyond the findings of earlier reviews (e.g. [1, 2, 13, 17, 20] this latest meta-analysis adds new information showing that education has a positive effect on hospital falls rates and risk. Large randomised trials [7, 57] showed the benefits of engaging patients and clinicians in education and training, in agreement with a recent scoping review by Heng [12]. In contrast, our meta-analysis found no evidence of falls reduction using scored falls risk screening tools, bed alarms, chair alarms or sensors.

Select studies of multi-factorial interventions showed favourable effects on hospital falls rates. These included combinations of at least two interventions such as patient or staff education, procedures around nurse handover, fast responses to call buttons, regular toileting, environmental modifications, assistive devices, exercise therapies, safe footwear, medication management, diet or management of cognitive impairment. The most favourable result for a multi-factorial RCT was by Stenvall *et al.* [40]. This trial had a low risk of bias and included rehabilitation, staff education, changes to systems, policies and procedures, diet and environmental modifications. However, patients allocated to the intervention arm of this study were cared for on a ward that had fewer beds (24 versus 27), yet 1.7 more full time equivalent allied health staff. Therefore, the importance of the falls interventions versus the enhanced staffing level was unclear.

Another multi-factorial RCT by Haines *et al.* [34] showed favourable results. They evaluated the targeted provision of therapeutic exercises, patient education, risk alert signs and hip protectors. In contrast, a high quality, cluster RCT by Barker *et al.* [5] that compared usual care with a nurse-led ‘6-PACK’ program did not find superior results for a multi-factorial approach to falls prevention. The 6-PACK program combined a FRAT with alert signs, bathroom supervision, a toileting program, clinician education, ensuring walking sticks and frames were in reach, low–low beds, bed and or chair alarms [5]. Several of the included interventions, such as use of low beds, FRATS and bed and/or chair alarms have limited or negative effects on hospital falls [45, 47, 48]. This contrasts with the beneficial effects reported for toileting programs [72], bathroom supervision [73] and other systems to mitigate falls [73].

A small number of individual studies reported a trend towards physiotherapy or additional rehabilitation therapies lowering hospital falls rates, however these were not significant [52, 55]. The GRADE analysis showed an overall low level of certainty for the results for the rehabilitation therapy trials included in the meta-analysis [52, 53, 56]. There was no evidence that low beds, bed alarms or chair alarms reduced hospital falls or injuries, in agreement with Oliver *et al.* [74]. In fact, our systematic review showed that for low beds and bed or chair alarms the rate of falls trended towards being higher [45, 48, 49].

There were several limitations of this analysis. We did not evaluate injury data associated with falls due to inconsistent definitions applied by studies. In addition, we did not examine the effects of care-giver education on falls risk or rates. It could be helpful to enlist family members as care providers to be part of the social network around patients, especially for patients with cognitive impairment, who have disproportionately high falls rates [75–78]. It was beyond the scope of this analysis to quantify the outcome of quality improvement (QI) initiatives, especially those pertaining to health professional education and training, despite growing traction of QI [76–78]. Preventing falls in real life clinical settings sometimes means iterative, locally driven QI with continuous monitoring of changes in local falls and injury data and health professional behaviours [79]. At an organisational level, hospital staff working on patient safety are interested in what works locally to produce sustained reductions in falls in addition to considering the gold-standard evidence afforded by global systematic reviews and meta-analyses [80]. In this regard education is arguably a powerful tool, although it is not clear which elements of educational design and delivery have the most optimal effects [7, 81]. The falls intervention taxonomy that we used differed to a small extent from Lamb [82], limiting direct comparisons with the prior Cochrane review. Finally, we did not review studies on falls in the home, community or residential aged care, given the different epidemiology related to falls and injuries in these environments [83–86]. Only studies published in English were included.

To conclude, no single definitive method exists for hospital falls prevention. This analysis of the global literature showed that education was the most effective strategy for reducing the rate and risk of hospital falls and multi-factorial interventions had a tendency to produce a positive impact.

## Supplementary Material

Supplementray_File_Figures_and_Tables_Morris_4_April_2022_afac077
