# Supplementary material for: Interventions to reduce falls in hospitals: a systematic review and meta-analysis
Source: Age Ageing. 2022 May 6;51(5):afac077. doi: 10.1093/ageing/afac077 (PMC9078046; doi:10.1093/ageing/afac077)
Supplement: Supplementray_File_Figures_and_Tables_Morris_4_April_2022_afac077 [file supplementray_file_figures_and_tables_morris_4_april_2022_afac077.docx]

**Supplementary File Figure 1. PRISMA flow diagram**

**Identification**

**Screening**

**Included**

**Eligibility**

Records identified through database searching
(n = 11,186)

Additional records identified through other sources
(n = 26)

Records identified
(n = 11,212)

Duplicates removed
(n = 3,006)

Records screened by title

(n = 8,206)

Records excluded
(n = 7,132)

Title and abstract assessed for eligibility

(n = 1,074)

Records excluded
(n = 844)

Full text articles assessed for eligibility

(n = 230)

Records excluded (n = 187)

**Reasons**

Not in hospital (n=45)

No or not usable falls data/wrong outcomes (n=52)

No comparison group (n=46)

Trial protocol/registration (n=9)

Abstract only (n=17)

Commentary/letter/review (n=8)

Summary of another study (n=8)

Thesis (n=2)

Studies included in review

(n = 43)

**Supplementary Figure 2. Risk of bias for randomized controlled trials for each domain**


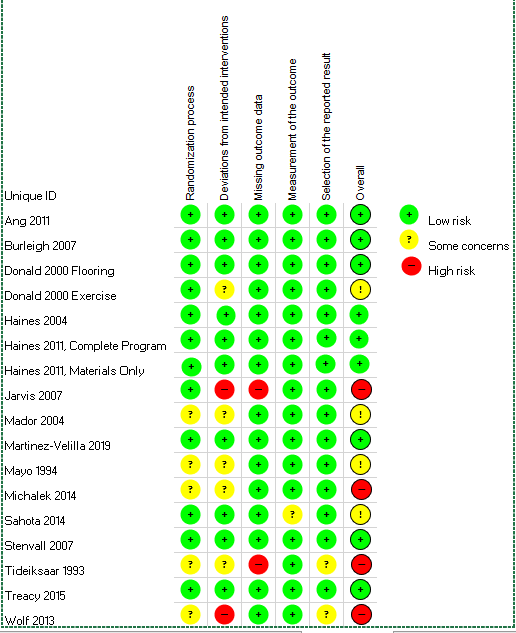


#### Supplementary Figure 3. Risk of bias for randomized controlled trials as a percentage

##### Supplementary File Table 1. Characteristics of included studies

|  | **Lead author (Year)** | **Country** | **Study design** | **Setting** | **Intervention participant details** | | | | **Control participant details** | | | | **Main Intervention Classification** | **Intervention frequency and/or duration** | **Falls specific outcomes** | **Falls recording methods** |
| --- | --- | --- | --- | --- | --- | --- | --- | --- | --- | --- | --- | --- | --- | --- | --- | --- |
|  |  |  |  |  | **Number (n)** | **Mean age (y)** | **Male (n)** | **Mean LOS (days)** | **Number (n)** | **Mean age (y)** | **Male (n)** | **Mean LOS (days)** |  |  |  |  |
| **1** | **Aizen (2015)** | Israel | Two-stage cluster-RCT | Geriatric rehabilitation | First Phase 200 | 84.6 | 108 | 19.66 | 308 | 84.1 | 135 | 24.7 | Multifactorial | Once a week over 6 months | Number of falls and fallers; Number of falls by risk; Falls per 1000 bed days; Falls hazard ratio | Incident reports, medical records, verbal nurse reports |
|  |  |  |  |  | Second Phase 244 | 80.8 | 73 | 23.9 |  |  |  |  |  |  |  |  |
| **2** | **Ang (2011)** | SG | RCT | Acute | 910 | 70.3 | 433 | NR | 912 | 69.7 | 472 | NR | Multifactorial | 30 min session per admission over 9 months | Number of falls; Relative risk estimate; Fall hazard ratio; Falls incidence and incidence rate | Electronic hospital occurrence report (eHOR) |
| **3** | **Barker (2016)** | Australia | Cluster RCT | Acute | 22670 | Median 68 | 11194 | Median 4 | 23575 | Median 67 | 12151 | Median 4 | Multifactorial | Once per admission, fall risk tool updated each nursing shift, over 12 months | Falls; Fallers; Injurious falls; Falls and fall injuries per 1000 patient days | Daily audit of medical records, verbal nurse reports, monthly audit of hospital incident reporting and other databases |
| **4** | **Bott (2019)** | USA | Case control quasi-experimental  pre-post design | Acute | 41 | 76.9 | 13 | NR | 54 | 76.22 | 30 | NR | Systems, policies and procedures | 3 months | Falls per 1000 patient days (presented as a ratio) | Hospital reports |
| **5** | **Burleigh (2007)** | UK | RCT | Acute and rehabilitation | 101 | 82.3 | 40 | Median 30 | 104 | 83.7 | 44 | Median 30 | Medication | For duration of patient LOS | Number of fallers, falls, and fractures | Falls accident forms |
| **6** | **Castro-Avila (2019)** | UK | Interrupted time-series analysis | NHS Trusts | 66 Trusts | NR | NR | NR | 84 | NR | NR | NR | Systems, policies and procedures | 51 months | Falls with harm | Monthly single-day census using the NHS safety thermometer |
| **7** | **Cumming (2008)** | Australia | Cluster RCT | Acute and geriatric rehabilitation | 2047 | 79.6 | 789 | Acute: 8.5; Rehab: 16.5 | 1952 | 78.4 | 834 | Acute: 8.2; Rehab: 16.8 | Multifactorial | For duration of patient LOS over 3 years | Number of falls, injurious falls and fractures | Incident reports, medical records, verbal nurse reports |
| **8** | **Daley (2020)** | US | Quasi-experimental | Neurological  and mixed general medical unit | NR | NR | NR | NR | NR | NR | NR | NR | Systems, policies and procedures | 3 months | Number of falls, Falls and fall injuries per 1000 patient days; other variables associated with fall rate | NR |
| **9a** | **Donald (2000) Environmental modification** | UK | RCT | Geriatric rehabilitation | 28 | 82.6 | 2 | 22.7 | 26 | 82.9 | 8 | 36.1 | Environmental modifications | 9 months | Incidence of falls; Relative risk of faller and falling | Accident report forms |
| **9b** | **Donald (2000) Exercise** | UK | RCT | Geriatric rehabilitation | 30 | 81.1 | 7 | 27 | 24 | 84.9 | 3 | 32 | Exercise | 9 months | Incidence of falls; Relative risk of falls | Accident report forms |
| **10** | **Drahota (2013)** | UK | Cluster RCT | Geriatric | 225 | 81.1 | 72 | Median 14 | 223 | 80.58 | 19 | Median 16 | Environmental modifications | 16 months | Injurious falls and falls per 1000 patient days; Fallers; Injury severity | Standardised forms |
| **11** | **Dykes (2010)** | US | Cluster RCT | Acute | 5160 | NR | 2335 | Median 3.25 | 5104 | NR | 2333 | Median 3.27 | Multifactorial | 6 months | Number of falls and repeat falls; Falls per 1000 patient days; Injurious falls | Event reporting system |
| **12** | **Haines (2004)** | Australia | RCT | Geriatric rehabilitation | 310 | 101 | 80 | 30 | 316 | 80 | 105 | 29 | Multifactorial | NR | Incidence rate of falls; Injurious falls; Proportion of participants who experienced one or more falls | Standardised incident report |
| **13** | **Haines (2010)** | Australia | Cluster RCT | Public hospitals | 9 wards | NR | NR | NR | 9 wards | NR | NR | NR | Assistive devices | 6 months | Falls per 1000 patient days per ward per month; Rate of injurious falls; Rate of falls resulting in head injury; Falls in bedroom per 1000 OBD per ward per month | Incident reporting system |
| **14a** | **Haines (2011) Complete program** | Australia | 3-group RCT | Acute and subacute | 401 | 75.3 | 185 | NR | 381 | 75.3 | 178 | NR | Education | Once per admission with one or more follow up sessions over duration of patient stay; 22 months | Falls and injurious falls per 1000 patient days; Proportion of patients who experienced 1 or more falls | Incident reporting system, medical notes, weekly patient interviews |
| **14b** | **Haines (2011) Materials only** | Australia | 3-group RCT | Acute and subacute | 424 | 74.7 | 201 | NR | 381 | 75.3 | 178 | NR | Education | Once per admission; 22 months | Falls and injurious falls per 1000 patient days; Proportion of patients who experienced 1 or more falls | Incident reporting system, medical notes, weekly patient interviews |
| **15** | **Hanger (2017)** | NZ | Prospective, observationa trial | Geriatric subacute | 100 | 83 | 43 | 24 | 78 | 83 | 33 | 24 | Environmental Modification | 31 months | Number of falls and injurious falls; Falls and injurious falls per 1000 bed days | Patient management system |
| **16** | **Hardin (2013)** | USA | Descriptive case-control | Acute | NR | NR | NR | NR | NR | NR | NR | NR | Systems  Including  Webcams | 6 months | Falls per 1000 patient days; Number of falls; Number of serious injuries | Site coordinators recorded data |
| **17** | **Healey (2004)** | UK | RCT | Acute and short-term geriatric rehabilitation | 905 | 81.4 | 362 | 18.31 | 749 | 81.2 | 300 | 21.29 | Multifactorial | 12 months | Number of falls and injurious falls; Falls and injurious falls per 1000 bed days; Relative risk of falls | Accident and incident reporting system |
| **18** | **Healey (2014)** | UK | Quasi-experimental | Acute | NR | NR | NR | NR | NR | NR | NR | NR | Multifactorial | 24 months | Falls and injurious falls per 1,000 patient days ; Relative risk of a fall | Incident reporting systems |
| **19** | **Hill (2015)** | Australia | Cluster RCT | Rehabilitation | 1623 | 81.4 | 624 | 12 | 1983 | 82.1 | 772 | 11 | Multifactorial | 30 min across 2-4 sessions | Falls and injurious falls per 1000 patient days; Proportion of falls, injurious falls and fallers | Hospital incident reports and patient note audits |
| **20** | **Jarvis (2007)** | UK | RCT | Rehabilitation | 14 | NR | 0 | NR | 15 | NR | 0 | NR | Exercise | Twice a day, five times a week during admission | Number of falls and fallers | Patient interviews |
| **21** | **Koh (2009)** | SG | Quasi-experimental | Acute | (Nurse data) 311 | 30.25 | 7 | NR | 278 | 34.35 | 1 | NR | Multifactorial | 15 months | Number of falls; Falls per 1000 patient bed days; Percentage of injury-associated falls | Fall incidence database, medical records |
| **22** | **Krauss (2008)** | USA | Quasi-experimental | Acute | NR | NR | NR | NR | NR | NR | NR | NR | Multifactorial | 9 months | Number of falls (assisted falls, toileting-related falls, repeat falls); Falls per 1000 patient days | Adverse event reporting system, medical records, verbal nurse reports |
| **23** | **Krepper (2014)** | USA | Two-group quasi-experimental | Acute | NR | NR | NR | NR | NR | NR | NR | NR | Systems policies and procedures | 6 months | Falls per 1000 patient days | Hospital reporting system |
| **24** | **Mador (2004)** | Australia | RCT | Acute | 36 | 82.1 | 21 | Median 17.5 | 35 | 82.9 | 16 | Median 17 | Multifactorial | 11 months | Number of fallers | Database of critical incidents |
| **25** | **Malfait**  **(2020)** | Belgium | Multi-centre matched controlled longitudinal design | Acute and  subacute | T0 177 | 64.7 | 69 | 20.9 | 92 | 63.5 | 46 | 42.9 | Systems policies and procedures | 22 months | Number of falls | Medical records, hospital incident reporting system |
|  |  |  |  |  | T1 168 | 65.1 | 77 | 22.0 | 98 | 61.8 | 53 | 44.8 |  |  |  |  |
|  |  |  |  |  | T2 164 | 65.9 | 59 | 17.7 | 75 | 63.4 | 41 | 44.5 |  |  |  |  |
| **26** | **Martínez-Velilla (2018)** | Spain | RCT | Acute | 185 | 87.6 | 76 | Median 8 | 185 | 87.1 | 85 | Median 8 | Exercise | 20-minute session twice daily between 5 to 7 days; 31 months | Proportion of fallers | NR |
| **27** | **Mayo (1994)** | Canada | RCT | Sub-acute | 65 | 70.9 | 34 | 75.5 | 69 | 72.9 | 38 | 67.2 | Assistive Device:  Identification bracelet | 11 months | Number and proportion of falls and fallers; Frequency of falls | Incident reports |
| **28** | **Michalek (2014)** | Germany | RCT | Acute geriatric | 58 | Median 84 | NR | 22 | 56 | Median 83 | NR | 21 | Medication System | For duration of patient LOS over 6 months | Number of fallers; Falls per 1000 patient days | Hospital recording system |
| **29** | **Montejano-Lozoya (2020)** | Spain | Quasi-experimental | Acute | 303 | 69.7 | 159 | 10.9 | 278 | 66.8 | 135 | 13.7 | Systems, policies and procedures | 8 months, 3 phases. Data collection for 5 months | Incidence of falls | Nurse Assessment Registry |
| **30** | **Nedved (2012)** | USA | Retrospective quasi-experimental | Acute | NR | 57.9 | NR | 2.68 | NR | 53.9 | NR | 3.59 | Systems, policies and procedures | 11 months | Percentage of days with or without falls; Falls per 1000 patient days | NR |
| **31** | **Padula (2011)** | USA | Non-equivalent control group design | Acute | NR | NR | NR | NR | NR | NR | NR | NR | Exercise | 2-3 sessions daily per admission; 6 months | Number of falls and injurious falls; Falls per 1000 patient days | Incident reports |
| **32** | **Sahota (2014)** | UK | Pragmatic parallel-arm individual RCT | Acute | 918 | 84.2 | 405 | 13 | 921 | 84.9 | 432 | 11.6 | Assistive devices  (bed and bedside chair pressure sensors) | 26 months | Number of bedside falls and injurious bedside falls per 1000 bed days; Fear of falling; Number of fallers | Incident reporting forms |
| **33** | **Sheppard (2021)** | UK | Time-series | Acute and subacute | Total: 36 wards | NR | NR | NR | Total: 36 wards | NR | NR | NR | Systems, policies and procedures | 35 months | Falls per 1000 occupied bed days | Incident reporting system |
| **34** | **Shorr (2012)** | USA | Cluster RCT | Acute | 10761 | 59.6 | 4875 | NR | 16911 | 59.1 | 7813 | NR | Assistive devices  (bed alarms) | 18 months | Falls, injurious falls and number of fallers per 1000 patient days | Standardised data collection tool |
| **35** | **Schwendimann (2006)** | Switzerland | Quasi-experimental design | Acute and geriatric rehabilitation | 198 | 72.5 | 69 | 12.4 | 211 | 68.9 | 94 | 11 | Multifactorial | 4 months | Proportion of fallers and fallers with multiple falls; Falls per 1000 patient days; Number of falls and injurious falls; Timing and type of falls; Time until first fall | Falls incidence reports |
| **36** | **Stenvall (2007)** | Sweden | RCT | Acute | 102 | 82.3 | 28 | 28 | 97 | 82 | 23 | 38 | Multifactorial | 30 months | Number of falls per 1000 days; Number of falls; Number of fallers, fallers with injuries, fallers with dementia | Medical records |
| **37** | **Tideiksaar (1993)** | USA | Case-controlled | Acute geriatric | 35 | 84 | 5 | NR | 35 | 84 | 5 | NR | Assistive devices  (bed alarms) | 9 months | Number of falls | Incident reports |
| **38** | **Treacy (2015)** | Australia | RCT | Rehabilitation | 81 | 82.6 | 30 | 22.7 | 81 | 81.4 | 28 | 26.8 | Exercise | 6 one-hour classes over 2 weeks | Number of falls; Incidence rate ratio | Patient interviews, medical records |
| **39** | **van Gaal (2011)** | Netherlands | Cluster randomised trial | Acute wards and nursing homes | (Hosp.) 1081 | 66 | 511 | NR | 1120 | 67 | 474 | NR | Education | 14 months | Falls per patient week | Medical records |
| **40** | **Visvanathan (2021)** | Australia | Cluster step-wedged | Geriatric wards | 1244 | 84 | 528 | Median: 16 | 1995 | 81.9 | 921 | Median: 11 | Assistive devices  (wearable sensors) | 103 weeks | Falls and injurious falls per 1000 patient days, proportion of fallers | Incident reports and medical records |
| **41** | **Vassallo (2004)** | UK | Quasi-experimental | Subacute geriatric | 275 | 81.6 | 118 | 26.9 | 550 | 82 | 176 | 21.3 | Multifactorial | During each patient admission | Number of falls, fallers, recurrent fallers and patients sustaining injury; Falls per patient days | Incident reporting system |
| **42** | **Wald (2011)** | USA | Quasi-randomised controlled trial | Acute | 122 | 80.5 | 58 | 3.4 | 95 | 80.7 | 39 | 3.1 | Multifactorial | 6 months | Falls per 1000 patient days | Incident reporting system |
| **43** | **Wolf (2013)** | Germany | Pilot RCT | Geriatric wards | 48 | NR | NR | NR | 50 | NR | NR | NR | Assistive devices  (bed alarms) | 13 months | Number of falls and fallers | Medical records |

USA = United States of America; UK = United Kingdom; SG = Singapore; NZ = New Zealand; RCT = Randomised controlled trial; NR = Not Reported; LOS = Length of stay; NHS = National Health Service

T0= baseline, T1= 3 months after baseline, T2= 9 months after baseline

Supplementary File Table 2. Cluster RCT Cochrane risk of bias

| **Author** | **Randomization** | **Identification and Recruitment** | **Deviations** | **Missing outcome data** | **Measurement of the outcome** | **Selection of reported results** | **Overall Bias** |
| --- | --- | --- | --- | --- | --- | --- | --- |
| Aizen 2015 | Some concerns | Low | Some concerns | Low | Low | Low | Some concerns |
| Barker 2016 | Low | Low | Low | Low | Low | Low | Low |
| Cumming 2008 | Low | Low | Low | Low | High | Low | Some concerns |
| Drahota 2013 | Some concerns | Some concerns | High | Low | Low | Low | Some concerns |
| Dykes 2010 | Low | Low | Low | Low | Low | Low | Low |
| Hardin 2013 | Low | Low | Low | Low | Some Concerns | Low | Some Concerns |
| Haines 2010 | Low | Low | Low | Low | Low | Low | Low |
| Healey 2004 | Some concerns | Low | Low | Some concerns | Low | Low | Some concerns |
| Healey 2014 | Some concerns | Low | Some concerns | Low | Low | Low | Some concerns |
| Hill 2015 | Low | Low | Low | Low | Low | Low | Low |
| Sheppard 2021 | High | High | High | Low | High | Some concerns | High |
| Shorr 2012 | Low | Low | Low | Low | Low | Low | Low |
| Van Gaal 2011 | Low | Low | Low | Low | Low | Low | Low |

Supplementary File Table 3. Method quality appraisal for non-randomised trials

| **Quasi-experimental study (JBI Appraisal Tool) ^24^** | | | | | | | | | | | |
| --- | --- | --- | --- | --- | --- | --- | --- | --- | --- | --- | --- |
| Author | Cause effect | Participants similar | Comparisons similar | Control group | Multiple measures | Follow-up | Consistent measurement | Reliable measurement | Statistical analysis | **Score /9** |  |
| Bott 2019 | Y | N | Y | Y | Y | N | Y | Y | N | **6** |  |
| Malfait 2020 | Y | N | Y | Y | Y | Y | Y | Y | Y | **8** |  |
| Castro-Avila 2019 | Y | N | N | Y | Y | Y | Y | Y | Y | **7** |  |
| Daley 2020 | Y | N | Y | Y | N | N | Y | Y | Y | **7** |  |
| Hanger 2017 | Y | Y | Y | Y | N | N | Y | Y | Y | **7** |  |
| Koh 2009 | Y | Y | Y | Y | Y | Y | Y | Y | Y | **9** |  |
| Krauss 2008 | Y | Y | Y | Y | Y | N | Y | Y | Y | **8** |  |
| Krepper 2014 | Y | Y | Y | Y | N | N | Y | Y | Y | **8** |  |
| Montejano Lozoya 2020 | Y | N | Y | Y | N | N | Y | Y | Y | **6** |  |
| Nedved 2012 | Y | N | Y | Y | Y | Y | Y | Y | Y | **8** |  |
| Padula 2011 | Y | N | Y | Y | N | Y | Y | Y | N | **6** |  |
| Schwendimann 2006 | Y | Y | Y | Y | N | Y | Y | Y | Y | **8** |  |
| Vassallo 2004 | Y | N | Y | Y | N | Y | Y | Y | Y | **8** |  |
| Visvanathan 2021 | Y | Y | N | N | Y | Y | Y | Y | Y | **7** |  |
| Wald 2011 | Y | Y | Y | Y | N | Y | Y | Y | Y | **8** |  |

Legend: Y=yes N=no or unclear

Supplementary File Table 4. Components of multi-element interventions

| **Lead author (Year)** | **Environment modification** | **Assistive devices** | **Patient education** | **Staff education** | **Falls risk reminders** | **Toileting** | **Medication** | **Exercise** | **Falls risk assessment** | **Care plan** | **Rounding** | **Nutrition** | **Systems, policies, procedures** |
| --- | --- | --- | --- | --- | --- | --- | --- | --- | --- | --- | --- | --- | --- |
| Aizen (2015) | 🗸 | 🗸 | 🗸 | 🗸 | X | X | X | X | 🗸 | 🗸 | X | X | X |
| Ang (2011) | X | X | 🗸 | X | X | X | X | X | X | X | X | X | X |
| Barker (2016) | ✓ | 🗸 | X | X | 🗸 | 🗸 | X | X | 🗸 | X | X | X | X |
| Cumming (2008) | ✓ | ✓ | ✓ | ✓ | X | X | ✓ | ✓ | ✓ | X | X | X | X |
| Dykes (2010) | X | ✓ | ✓ | X | ✓ | X | X | X | ✓ | ✓ | X | X | X |
| Haines (2004) | X | ✓ | ✓ | ✓ | ✓ | X | X | ✓ | ✓ | X | X | X | X |
| Healey (2004) | 🗸 | 🗸 | X | X | X | X | 🗸 | X | 🗸 | 🗸 | X | X | X |
| Healey (2014) | X | X | X | X | X | X | ✓ | X | ✓ | X | ✓ | X | X |
| Hill (2015) | X | X | ✓ | ✓ | ✓ | X | X | X | X | X | X | X | X |
| Koh (2009) | X | X | X | 🗸 | 🗸 | X | X | X | 🗸 | X | X | X | 🗸 |
| Krauss (2008) | X | X | 🗸 | 🗸 | 🗸 | 🗸 | 🗸 | X | X | X | X | X | X |
| Mador (2004) | X | X | X | 🗸 | X | X | X | X | X | 🗸 | 🗸 | X | X |
| Schwendimann (2006) | ✓ | ✓ | X | ✓ | 🗸 | 🗸 | X | 🗸 | 🗸 | X | ✓ | X | X |
| Stenvall (2007) | X | X | X | ✓ | X | X | 🗸 | ✓ | X | ✓ | X | 🗸 | X |
| Vassallo (2004) | ✓ | ✓ | 🗸 | X | ✓ | X | 🗸 | X | ✓ | 🗸 | X | X | X |
| Wald (2011) | X | X | X | 🗸 | X | X | X | X | 🗸 | 🗸 | X | X | X |

###### Supplementary File Table 5. GRADE evidence summaries

**3a Education compared to usual care for preventing falls in hospitals**

**Author(s)**: RaR and OR: Haines et al. (2011), Hill et al. (2015)

| N studies | Study design | Risk of bias | Inconsistency | Indirectness | Imprecision | Other considerations | Certainty |
| --- | --- | --- | --- | --- | --- | --- | --- |
| Rate of falls | | | | | | | |
| 2 | Randomised trials | Not serious | Not serious | Not serious | Not serious | None | ⨁⨁⨁⨁ HIGH |
| Odds of falling | | | | | | | |
| 2 | Randomised trials | Not serious | Not serious | Not serious | Not serious | None | ⨁⨁⨁⨁ HIGH |

**3b Assistive devices compared to usual care for preventing falls**

**Author(s)**: RaR: Haines et al. (2010), Mayo et al. (1994), Sahota et al. (2014), Shorr et al. (2012), OR: Mayo et al. (1994), Sahota et al. (2014), Wolf et al. (2013),

| N studies | Study design | Risk of bias | Inconsistency | Indirectness | Imprecision | Other considerations | Certainty |
| --- | --- | --- | --- | --- | --- | --- | --- |
| Rate of falls | | | | | | | |
| 4 | Randomised trials | Serious^a^ | Not serious | Not serious | Not serious | None | ⨁⨁⨁◯ MODERATE |
| Odds of falling | | | | | | | |
| 3 | Randomised trials | Very serious^b^ | Not serious | Not serious | Not serious | None | ⨁⨁◯◯ LOW |

#### Explanations

a. Risk of bias downgraded due to 2/4 studies rated as having "some concerns"

b. Risk of bias downgraded due to 2/3 studies rated as having "some concerns" and one study rated as having "high" concerns

**3c Rehabilitation therapies compared to usual care for preventing falls in hospitals**

**Author(s)**: OR: Donald et al. (2000), Jarvis et al. (2007), Martinez-Vellila et al. (2018)

| N studies | Study design | Risk of bias | Inconsistency | Indirectness | Imprecision | Other considerations | Certainty |
| --- | --- | --- | --- | --- | --- | --- | --- |
| **Odds of falling** | | | | | | | |
| 3 | Randomised trials | Very serious^a^ | Serious^b^ | Not serious | Serious^c^ | None | ⨁◯◯◯ VERY LOW |

#### Explanations

a. Risk of bias downgraded due to 1/3 studies rated as having "some concerns" and 1/3 studies rated as "high"

b. Inconsistency due to large I2 (62%)

c. Imprecision downgraded due to 1/3 studies having wide confidence intervals (0.59, 44.12)

**3d Environmental modifications compared to usual care for preventing falls in hospitals**

**Author(s)**: OR: Donald et al. (2000) flooring, Drahota et al. (2013)

| N studies | Study design | Risk of bias | Inconsistency | Indirectness | Imprecision | Other considerations | Certainty |
| --- | --- | --- | --- | --- | --- | --- | --- |
| **Odds of falling** | | | | | | | |
| 2 | Randomised trials | Serious^a^ | Serious^b^ | Not serious | Very serious^c^ | None | ⨁◯◯◯ VERY LOW |

#### Explanations

a. Risk of bias downgraded because 1/2 studies were scored as having "some concerns"

b. Inconsistency downgraded due to large I2 (40%)

c. Imprecision downgraded due to very wide confidence intervals in 1/2 studies (0.95, 73.37)

**3e Multifactorial interventions compared to usual care for preventing falls in hospitals**

**Author(s)**: RaR: Barker et al. (2016), Cumming et al. (2008), Haines et al. (2004), Stenvall eta al. (2007), Van Gaal et al. (2011) OR Non- RCT: Schwendimann et al. (2006), Vassallo et al. (2004):

OR RCT: Aizen et al. (2015), Ang et al. (2011), Barker et al. (2016), Cumming et al. (2008), Dykes et al. (2010), Haines et al. (2004), Mador et al. (2004), Stenvall et al. (2007)

| N studies | Study design | Risk of bias | Inconsistency | Indirectness | Imprecision | Other considerations | Certainty |
| --- | --- | --- | --- | --- | --- | --- | --- |
| **Rate of falls (RCT’s)** | | | | | | | |
| 6 | Randomised trials | Serious^a^ | Serious^b^ | Serious^c^ | Not serious | None | ⨁◯◯◯ VERY LOW |
| **Odds of falling (RCT’s)** | | | | | | | |
| 8 | Randomised trials | Serious^d^ | Not serious | Serious^e^ | Very serious^f^ | None | ⨁◯◯◯ VERY LOW |
| **Odds of falling (Non RCT’s)** | | | | | | | |
| 2 | Observational studies | Not serious | Not serious | Not serious | Serious^g^ | None | ⨁◯◯◯ VERY LOW |

#### Explanations

a. Risk of bias downgraded due to 1/6 studies rated as having "some concerns"

b. Inconsistency downgraded due to large I2 (49%)

c. Indirectness downgraded due to 1/6 studies conducted on a population with "femoral neck fracture"

d. Risk of bias downgraded due to 4/8 studies rated as having "some concerns"

e. Indirectness downgraded due to 1/8 studies conducted on a population with "femoral neck fracture"

f. Imprecision downgraded due to very wide confidence intervals across 4/8 studies with 1/4 studies being extremely large (0.03, 26.35), (0.17, 39.01), (0.83, 10.65), (0.00, 4147.42)

g. Imprecision downgraded due to wide confidence intervals in 1/2 studies (0.05, 22.09)

Supplementary File Table 6. Risk of Bias for studies assessing disinvestment from scored FRATs

| **Author** | **Randomization** | **Identification and Recruitment** | **Deviations** | **Missing outcome data** | **Measurement of the outcome** | **Selection of reported results** | **Overall Bias** |
| --- | --- | --- | --- | --- | --- | --- | --- |
| Morris et al. (2021) | Low | Low | Low | Low | Low | Low | Low |
| Jellett et al. (2020) | Low | Low | Low | Low | Low | Low | Low |

**Supplementary File Table 7: CERT results**

| CERT item | Cumming et al. 2008 | Donald et al. 2000 | Haines et al. 2004 | Jarvis et al. 2007 | Martinez-Velilla et al. 2019 | Padula ae al. 2011 | Schwendimann at al. 2006 | Stenvall et al. 2007 | Treacy et al. 2015 | **TOTAL** |
| --- | --- | --- | --- | --- | --- | --- | --- | --- | --- | --- |
| 1. Equipment | X | X | **✓** | X | **✓** | X | X | X | **✓** | **3** |
| 2. Qualifications of instructor | **✓** | **✓** | **✓** | **✓** | **✓** | **✓** | **✓** | **✓** | **✓** | **9** |
| 3. Individual or Group | **✓** | X | **✓** | X | **✓** | X | X | X | **✓** | **4** |
| 4. Supervision | **✓** | **✓** | **✓** | **✓** | **✓** | **✓** | X | **✓** | **✓** | **8** |
| 5. Adherence | X | X | X | X | **✓** | X | X | X | **✓** | **2** |
| 6. Motivation | X | X | X | X | X | X | X | X | X | **0** |
| 7a. Progression rule | X | X | X | X | X | X | X | X | **✓** | **1** |
| 7b. Progression described | X | X | X | X | **✓** | X | X | X | **✓** | **2** |
| 8. Exercise detail | X | **✓** | **✓** | X | **✓** | X | X | X | **✓** | **4** |
| 9. Home program | X | X | **✓** | X | X | X | X | X | **✓** | **2** |
| 10. Non-exercise components | **✓** | **✓** | **✓** | X | X | X | **✓** | **✓** | X | **5** |
| 11. Adverse events | **✓** | **✓** | **✓** | **✓** | **✓** | **✓** | **✓** | **✓** | **✓** | **9** |
| 12. Setting | **✓** | **✓** | **✓** | **✓** | **✓** | **✓** | **✓** | **✓** | **✓** | **9** |
| 13. Intervention described | X | **✓** | **✓** | X | **✓** | X | X | **✓** | **✓** | **5** |
| 14a. Generic or tailored | **✓** | **✓** | **✓** | **✓** | **✓** | **✓** | X | X | **✓** | **7** |
| 14b.Tailoring method | X | X | X | X | **✓** | X | X | X | X | **1** |
| 15. Starting level | X | X | X | X | **✓** | X | X | X | X | **1** |
| 16a. Fidelity measure | X | X | X | X | **✓** | X | X | X | **✓** | **2** |
| 16b. Fidelity described | **✓** | **✓** | X | X | X | X | X | X | **✓** | **3** |
| **Study score xx/19** | **8** | **9** | **11** | **5** | **14** | **5** | **4** | **6** | **15** |  |

**Legend: ✓** reported CERT item **X** did not report CERT item
